# Use of agro-industrial by-products containing tannins for the integrated control of gastrointestinal nematodes in ruminants

**DOI:** 10.1051/parasite/2022010

**Published:** 2022-02-28

**Authors:** Hervé Hoste, Griselda Meza-OCampos, Sarah Marchand, Smaragda Sotiraki, Katerina Sarasti, Berit M. Blomstrand, Andrew R. Williams, Stig M. Thamsborg, Spiridoula Athanasiadou, Heidi L. Enemark, Juan Felipe Torres Acosta, Gabriella Mancilla-Montelongo, Carlos Sandoval Castro, Livio M. Costa-Junior, Helder Louvandini, Dauana Mesquita Sousa, Juha-Pekka Salminen, Maarit Karonen, Marika Engstrom, Johannes Charlier, Vincent Niderkorn, Eric R. Morgan

**Affiliations:** 1 Université de Toulouse, UMR 1225 IHAP INRAE/ENVT 31076 Toulouse France; 2 HAO/DEMETER, Campus Thermi 57001 Thessaloniki Greece; 3 Norwegian Centre for Organic Agriculture 6627 Tingvoll Norway; 4 University of Copenhagen 1870 Frederiksberg C Denmark; 5 Scotland’s Rural College (SRUC), Easter Bush EH25 9RG United Kingdom; 6 Norwegian Veterinary Institute 1400 Ås Norway; 7 CONACYT-Facultad de Medicina Veterinaria y Zootecnia, Universidad Autónoma de Yucatán Carretera Mérida-Xmatkuil km 15.5 Mérida Yucatán 97000 México; 8 Laboratório de Controle de Parasitos, Centro de Ciências Biológicas e da Saúde, Departamento de Patologia, Universidade Federal do Maranhão, São Luis Maranhão MA 65080-805 Brazil; 9 Laboratório de Nutrição Animal, Centro de Energia Nuclear na Agricultura, Universidade de São Paulo 13400-970 Piracicaba São Paulo Brazil; 10 Natural Chemistry Research Group, University of Turku 20014 Turku Finland; 11 Kreavet Hendrik Mertensstraat 17 9150 Kruibeke Belgium; 12 Université Clermont Auvergne, INRAE, VetAgro Sup, UMR Herbivores 63122 Saint-Genes Champanelle France; 13 School of Biological Sciences, Queens University, Belfast BT9 5DL Northern Ireland United Kingdom

**Keywords:** Plant secondary metabolites, Plant specialised metabolites, Tannins, Anthelmintic resistance, Gastrointestinal nematodes, Circular economy

## Abstract

Previous studies have illustrated that different bioactive legume fodders containing condensed tannins might represent one of the options for integrated sustainable control of gastrointestinal nematodes (GIN) in ruminants, which may help address the worldwide development of resistance to synthetic anthelmintics. More recently, impetus has been given to assess the potential antiparasitic activity of less conventional resources, represented by different agro-industrial by-products (AIBPs). This review presents *in vitro* and *in vivo* results obtained with a range of tannin-containing AIBPs of various geographical and botanical origins, namely AIBP of nuts, temperate and tropical barks, carob, coffee and cocoa. They tend to confirm the “proof of concept” for their antiparasitic effects and also for other aspects of ruminant production in an agro-ecological context. Socio-economic aspects of the exploitation of such non-conventional resources are also discussed as potential models of the circular economy, by using waste. The different modes of use of these resources are presented in this review, as well as strengths, weaknesses, opportunities, and threats (SWOT) analyses to illustrate the advantages and limitations of on-farm use.

## Abstract (fr)

Plusieurs études antérieures ont illustré le fait que des légumineuses bioactives contenant des tannins condensés peuvent représenter une des alternatives à intégrer avec d’autres options pour une maitrise durable des nématodes gastro-intestinaux en réponse au développement constant et à l’expansion continue à l’échelle mondiale des résistances aux anthelminthiques de synthèse. Des recherches plus récentes se sont intéressées au potentiel d’application de ressources moins conventionnelles que représentent des coproduits agroindustriels (CPAI). Cette revue vise à présenter des résultats *in vitro* et *in vivo* obtenus avec une gamme de CPAI d’origines géographiques et botaniques diversifiées (coproduits de l’industrie des noix, du bois (en régions tempérées et tropicales), du caroubier, du café et du cacao). Ces résultats ont confirmé la preuve de concept pour les effets antiparasitaires, et aussi pour d’autres volets de la production des ruminants dans un contexte agro écologique de l’élevage. Par ailleurs, les aspects socio-économiques d’exploitation de ces ressources, considérées jusqu’à présent comme des déchets, dans un modèle de circuits courts sont aussi évoqués. Les avantages et inconvénients des différentes modalités d’exploitation des CPAI sont aussi discutés dans le cadre d’une analyse SWOT.

## Context

Worldwide, infection of the gastrointestinal tract of ruminants with parasitic nematodes remains the main challenge associated with outdoor production. This is because of the consequences on animal health and welfare of these helminth infections and also because of their major impact on productivity and on the economy of farms [22, 23]. The efficient control of these infections is essential to improve livestock health and production and consequently rural livelihoods.

For several decades, the control of these parasitic infections has mainly relied on the repeated use of chemical anthelmintics (AHs) provided by pharmaceutical companies. However, the accelerating development of resistance to AHs in worm populations and the extent of the phenomenon, including the rise of multi-resistant isolates, has been reported worldwide [102], see also STAR IDAZ website https://www.star-idaz.net/priority-topic/helminths-including-anthelmintic-resistance/ and the website of the COST action COMBAR https://www.combar-ca.eu/). Moreover, the development of resistance to AHs and other chemical drugs appears to be an inevitable process [119]. These different facts underline the need for a change in the mode of control of Gastrointestinal nematodes (GIN), instead of exclusive reliance on synthetic AH drugs. The general objective is nowadays to promote a more integrated approach, based on a combination of options which correspond to different principles and modes of action [112]. This more sustainable approach, which was foreseen even more than 20 years ago [110], is now generally agreed among parasitologists to be essential [22, 78].

Within the “basket of options” to control GIN [55], research into bioactive plants with AH properties has received strong impetus since the end of the 1990s. These antiparasitic activities have been associated with the presence of a range of plant secondary metabolites (= plant specialised metabolites) (PSMs) which are present in a large number of botanical families. For example, the potential use of chicory (*Cichorium intybus*) for its AH properties has been related to the presence of sesquiterpene lactones [92], while proteinases have been considered responsible for anthelmintic activity in several tropical plants [109]. Moreover, some fodder species of the Legume family (Fabaceae) containing condensed tannins (CTs) have been used as models to explore the AH effects of this particular class of PSM [45], e.g. sulla (*Hedysarum coronarium*), big trefoil and bird’s-foot trefoil (*Lotus pedunculatus* and *L. corniculatus*), sainfoin (*Onobrychis viciifolia*) and sericea lespedeza (*Lespedeza cuneata*). Data on evaluation of PSMs against parasites have generally relied first on a range of *in vitro* assays, followed by studies performed in animals, either in controlled experimental conditions or in controlled systematic trials. A last step is to confirm the possible application by studies based on on-farm conditions [47].

The main conclusions from these studies in CT-containing legumes, working hypotheses on the mode of action, and bottlenecks or hurdles identified from the various experimental studies are:

- the presence of CT-containing forages in the ruminant diet can affect the dynamics of GIN infections by disrupting the biology of different key stages of the GIN life cycle;
- the antiparasitic activity has been associated with the presence of CTs and also other related polyphenols, such as flavonoids, that are biosynthetic precursors of CTs;
- in regard to the potential application of such bioactive resources in farming conditions, the results have led to propose the concept of “nutraceuticals”. These are resources combining nutritional and health values for ruminants [47]. In contrast to synthetic AH drugs, nutraceutical fodders are not imposed (administered by force) but proposed to the animals. Therefore, the AH effects of such CT-containing resources used as nutraceuticals depend first on voluntary feed intake (VFI) by sheep, goats or cattle. Torres Acosta and his group [112] have proposed criteria to define nutraceuticals with antiparasitic activity;
- based on the “direct” hypothesis, relying on pharmacological-like effects of CT on the different key stages of GIN [45], data have been obtained to suggest that the *in vivo* AH effects depend on both a sufficient length of time to distribute the bioactive resources to animals, as well as a sufficient level (threshold) of polyphenols in the ruminants’ diet to expose worms to the bioactive compounds in the relevant digestive organs.

The identification of a main role of CTs and related polyphenols in the AH activity of legume forages has stimulated further exploration of the potential activity of other resources [46]. In particular, the worldwide coexistence of GIN in livestock and of polyphenol-containing legumes led to further studies to examine the value of a range of tropical plants, especially of the Fabaceae family, under a wide range of environments and management conditions. Secondly, the presence of polyphenolic compounds in a wide range of agro-industrial by-products (AIBPs) has stimulated interest in the potential antiparasitic activity of these resources.

**Figure 1:**
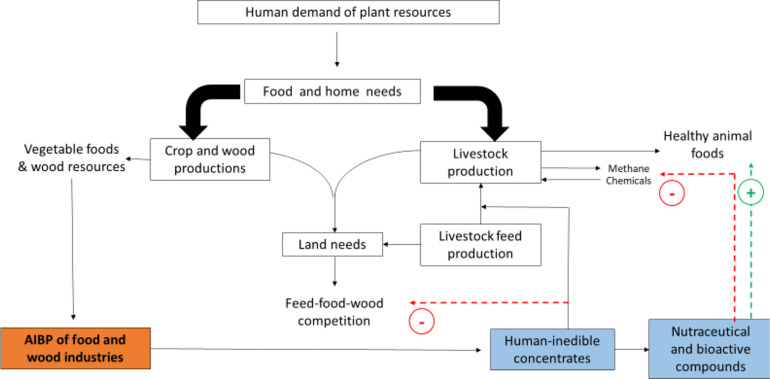
Beneficial role of the use of agro-industrial by-products (AIBPs) in animal production**.** A framework to develop the circular economy, including the potential effects on animal health through the use of nutraceuticals (Adapted from [26]).

The early impetus to exploit different AIBPs has been influenced by their potential nutritional value, and has focused on by-products of the food and wine industries, e.g. wine grape pomace, citrus pulps, and oil cakes. This first valorisation has been identified since the 1960’s and has led to long-term use of AIBPs as feeding resources in different livestock species, including ruminants. More recently, there has been renewed interest to exploit these AIBPs because of the presence of bioactive components. The PSMs contained in AIBPs can help to address several novel objectives. For instance, phasing-out of growth promoters and antibiotics, and a move towards the concept of agroecology in order to achieve more sustainable management of agriculture and livestock breeding, according to different criteria in a holistic framework, as illustrated in Figure 1.

*The general objective* of this review is to present a critical overview of the potential application of AIBPs by focusing on tannin-containing resources and on their combined nutritional and health-promoting value as nutraceuticals, specifically with potential AH activities against GIN of ruminants. Instead of being exhaustive, this review will focus on a few selected models of AIBPs obtained from different agro-industries, in order to illustrate the diversity of AIBPs, which can be exploited because of their bioactivity across a wide range of environmental conditions. These also exemplify a model of the circular economy to exploit industrial waste, addressing economic and social issues and enhancing the sustainability of food production [26]. For each selected example of an AIBP, data will be provided to illustrate:

- the geographical, historical, and economic context of each type of production,
- the potential AH activity and other antiparasitic effects (e.g. on other infections),
- a critical evaluation of the phytochemical compounds whose role is suspected to underpin the AH effects,
- the different possibilities of utilising these AIBPs in current livestock production systems.

Furthermore, besides their antiparasitic effects, utilising the bioactivity of AIBPs in an agro-ecological context could add wider value to ruminant production and nutrition. These are also discussed, as well as potential further implementation in other livestock species (pigs, horses, poultry, and rabbits) and parasites. An analysis of the strengths, weaknesses, opportunities and threats (SWOT) surrounding the application of AIBPs in this context is also presented, against a wide range of criteria (e.g. availability, cost, competition with other industries, and other possible uses in animal health) for the AIBPs presented in this review.

## AIBPs useful for parasite control

### Temperate nuts: chestnuts and hazelnuts

Nuts are fruits composed of an edible seed (called “almonds”) and of hard shells which are non-edible and which correspond to the pericarps composed of three separate layers. Hazelnut (*Corylus avellana,* Betulaceae) and chestnut (*Castanea sativa,* Fabaceae) form the basis of important industries in southern Europe, China and Central Asia, the USA (for hazelnuts) and South America (for chestnuts). In both cases, the almonds are of high value for human consumption. However, the hard shell, which is composed of the endocarp-meso and exocarps, are usually considered waste, although they might represent almost 15–20% of the whole weight of the fruit (e.g. for chestnut). The by-product (BP) of hazelnuts consists only of the endocarps, while for chestnuts, the BP constitutes a mixture of endocarps and mesocarps.

Leaves of nut-producing and other trees also have potential uses. The presence of tannins in the barks of different trees (e.g. oak trees, chestnut trees) of the Fagaceae family have been empirically exploited in Europe since early ancient times in tanneries for leather production. In addition, a study based on different *in vitro* assays [88] confirmed the potential AH effects of extracts of leaves of hazelnut and oak (*Quercus robur*) on three major GIN genera of ruminants. These early results, combined with the presence of condensed tannins in both hazelnuts and chestnut peels, helped to explain the bioactivity of the BP of nut peels and their potential antiparasitic agents.

For hazelnut and chestnut, several *in vitro* studies have indicated potential benefits of including the BP in ruminant feed, and for future use as nutraceuticals [47]. Various *in vitro* assays have been performed, with *Haemonchus contortus* the most common GIN model species used [66], although effects on the intestinal species *Trichostrongylus colubriformis* have also been examined [9, 30]. Moreover, the hypothesis of a suspected major role of tannins and other polyphenols in observed antiparasitic effects was confirmed by the use of tannin inhibitors such as polyethylene glycol (PEG) or polyvinylpolypyrrolidone (PVPP) [9, 66, 88].

In some of these *in vitro* studies, sainfoin extracts were included in the experimental design, thus allowing a comparison of values of half-maximal inhibitory concentrations (IC_50_) with hazelnut [13, 98] and chestnut extracts. Overall, lower IC_50_ values for the BP of nuts were obtained, suggesting higher AH activity than sainfoin, which is used as a reference of a tanniferous legume [47]. For example, the IC_50_ obtained with an acetone/aqueous extract defined on larval exsheathment inhibition ranged from 47 to 104 μg/mL for hazelnut and 99–399 μg/mL for sainfoin [13]. Similar values of half-maximal effective concentration (EC_50_) were obtained with chestnut extracts (31–96 μg/mL) [30]. These studies also explored the variability in AH efficacy for both hazelnut and chestnut depending on a range of factors, namely the environmental conditions of production (e.g. chestnuts grown in France or Chile), genetic factors (cultivars), and in the case of chestnuts, different technological processes applied to isolate the almonds (= fruits/seeds).

For both types of BP, AH properties have been confirmed *in vivo*. When hazelnut peels were distributed to goats experimentally infected with *H. contortus* and *T. colubriformis*, a significant reduction of > 50% in nematode faecal egg count (FEC) was observed, which was related to a decrease in the fecundity of female *Haemonchus.* A non-significant reduction of 45% in *T. colubriformis* populations was also found [34]. In a second step, the distribution of hazelnut peels in goats targeting the early phase of infection led to decreases of 48% for *H. contortus* and 42% for *T. colubriformis* in the establishment of third stage larvae of each species. One of the findings of the study was the ability of goats to voluntarily ingest up to 350 g of hazelnut peels per day, following a period of adaptation. According to the same design, similar results (decrease by 40% in FEC) were obtained in lambs with daily consumption of peels limited to 140 g [34].

The potential benefits of adding hazelnut peels to the diets of lambs infected with *H. contortus* was confirmed in another study, in which adding hazelnut peels to sainfoin pellets obtained significant decreases of 50–60% in egg excretion, but without any difference in worm numbers [40]. However, other studies [98] examining the AH consequences of prolonged feeding of hazelnut peels to infected lambs did not provoke any significant changes in egg excretion by *Haemonchus*, and only a small significant decrease in egg excretion and female fecundity for *T. colubriformis.* The value of combining tannins from two different resources was again illustrated in a recent study, which examined the AH effects when incorporating chestnut peels in sainfoin pellets offered to lambs infected with both abomasal and intestinal species [68]. With only tannins from chestnuts, the effects on egg excretion were non-significant. However, the combination of sainfoin and chestnut BP was associated with significant effects on the parasites and favourable consequences for host resilience, i.e. decreased anaemia.

The availability of tannins from the AIBP of chestnuts and hazelnuts has also been investigated, to better understand the mode of action of tannins on different GIN stages and/or species. Again, comparison with data obtained with CT containing legumes (e.g. sainfoin) is informative. The hypothesis of a direct mode of action of tannins against nematodes has been confirmed by results of several *in vitro* studies, including examination of structural changes based on observations using scanning and transmission electron microscopy [44, 45]. Results showed that changes occurred in the cuticle of adult worms, which were not seen after exposure to sainfoin extracts [69, 70].

Several studies have provided information not only on the content of tannins and other polyphenols, but also on the qualitative characterisation of both chestnut and hazelnut peels, namely the procyanidin to prodelphinidin ratio (PC/PD), the mean degree of polymerisation (mDP) and the cis/trans ratio [98]. Because some of these studies also included data on sainfoin, and other CT-containing fodders, and an evaluation of the AH effects, the results helped to address basic questions on the structure-AH activity relationship, and to analyse factors modulating the antiparasitic effects [79]. Comparison between a fodder (sainfoin) and hazelnut peel has also been performed to examine the distribution and fate of tannins from each plant in the sheep digestive tract [97].

Phytochemical analyses using ultra performance liquid chromatography-tandem mass spectrometry (UPLC-MS/MS) have been performed to characterise a variety of chestnut BPs of different geographical origins and following different technological processes (direct heat versus microwave). Results showed the presence of CT and both ellagic and gallic acids, suggesting the co-presence of some hydrolysable tannins. The characterisation of CT on eight samples suggested generally higher PD-% than PC-%, with PD content ranging from 46% to 84%. The mDP was 14.8, and the CT content ranged from 1.01 to 4.01 g per 100 g of dry matter (DM) (JP Salminen, personal communication).

For hazelnut peels, the main PSMs have been identified as being oligomeric and polymeric CT. Based on a method of thiolytic degradation coupled with high performance liquid chromatography (HPLC), the content of tannins in hazelnut peels was 6.27 g of proanthocyanindin per 100 g DM (compared with 1.71 in sainfoin). The CT of hazelnut peels consisted mostly of procyanidins (PC/PD ratio 72/28) with mDP of 13.3 [97]. Moreover, the CT of hazelnut endocarps was also characterised by a high level of galloylated flavonoids.

Compared with tannin containing legume fodders like sainfoin (*Onobrychis viciifolia*) or sericea lespedeza (*Lespedeza cuneata*), knowledge of the properties of hazelnut and chestnut BP remain limited. However, they have confirmed the potential of tannin-containing AIBP of nuts to aid in GIN control. The early results from chestnuts and hazelnuts have led to further studies using BP from other nut industries like shea nut (*Vitellaria paradoxa,* Sapotaceae) [100], peanut (*Arachis hypogaea*, Fabaceae) [62] and walnut (*Juglans* spp.*,* Juglandaceae).

### Mediterranean carob pods

Carob (*Ceratonia siliqua*) is a leguminous tree that is traditionally found in the Mediterranean area. Spain is the main producer and exporter, followed by Italy, Morocco, Portugal, Greece, Turkey and Cyprus. Cultivation also occurs in areas with similar climates, like the South-East USA, and parts of Australia and South Africa [10]. Carob fruit is a brown pod whose main raw material used in industry consists of the pulp (90% of pod weight) and the seeds. Carob pulp has a high sugar content (48–56%), and relatively low fat (0.4–0.8%) and protein (3–4%) [10, 67].

Carob trees have been used for centuries as a cheap source of both human and animal nutrition. Since they are mostly cultivated in dry areas with low rainfall, they require little attention and live up to 150 years [42, 67, 99]. Nowadays, carobs are used in food, pharmaceutical and cosmetic industries [54, 114] and in the fast-growing health food industry, since carob products have important nutritional and medicinal properties for humans [24, 106]. The BP of carob (CaBP) described herein concern the seedless carob fruit, called “pulp”, which is crushed and traditionally added to animal feed.

Carob tree parts (pods, seeds, germs, leaves and tree barks) are substantial sources of polyphenolic compounds, such as gallic acid, catechin, epicatechin, epicatechin gallate, epigallocatechin, epigallocatechin gallate, myricetin, and quercetin and their derivatives. Carob pod contains a higher amount of polyphenols in comparison to seed or germ [107]. Although a higher level of condensed than hydrolysable tannins is present, their reported concentration varies. Bravo et al. (1994) [14] and Batlle and Tous (1997) [10] reported concentrations of 16–20% w/w DM of CT in ripe carob pods. This has been debated by Priolo et al. (2000, 2002) [94, 95], who claimed that the pods have low content of CT, but exceptionally high biological activity. Silanikove et al. (2006) [106] demonstrated that the yield of CT is considerably affected by the extraction method applied (from 5.0 g/100 g DW with acidic methanol to 17.2% with urea-buffer solution), suggesting that carob pods are a rich source of CT. Similarly, Saratsi et al. (2020) [103] reported 5.84 g CT/100 g DW with acetone-HCl/butanol extraction method and 7.20 g CT/100 g DW based on the thiolysis method.

There is only one published *in vitro* study testing the AH efficacy of carob pods. In this study, the larval exsheathment inhibition assay (LEIA) was used to test the effect of CaBP against *H. contortus* and *T. colubriformis* larvae. The results showed no effect against *H. contortus*, but a noteworthy effect against *T. colubriformis.* Specifically, when larvae were exposed to an extract concentration of 1200 μg/mL, only 40.57% of L3 of *T. colubriformis* exsheathed, yielding an estimated EC_50_-value of 1 163.85 μg/mL [104].

Confirmation of the AH properties of CaBP was then obtained *in vivo*. CaBP meal was incorporated into the ration of sheep experimentally infected with *H. contortus* and *T. colubriformis*, and a significant reduction of > 39% in FEC was observed, which was related mainly to a decrease in egg output of females and to a lesser degree in the number of adult parasites. Moreover, the hypothesis of a suspected major role of tannins in these antiparasitic effects was confirmed by use of inhibitors such as PEG [103].

Particularly for *H. contortus*, it appears that fecundity is only affected when the worms are exposed to CT during maturation, and not when they are already mature adults. On the other hand, when CaBP was consumed for two weeks by animals in which adult worm populations were already established and patent, the main finding was a significant decrease in *H. contortus* worm counts. Overall, the current study adds further support to the observation that most of the CT effect is related to abomasal parasites, and not as much to small intestinal parasites [103]. In one of the above-mentioned trials, a combination of a fodder (sainfoin) and a BP (CaBP) was offered to animals to investigate whether different tannin sources can act synergistically. No synergistic effect was recorded [103]. In this study, the main tannins of carob were CT whose PC/PD ratio approximates to 3.3/96.7, and an mDP of 31.2 associated with a % galloylation close to 41.1. However, like for tannin-containing legumes, agro-environmental conditions have a strong effect on compositional traits, including condensed tannin composition [56].

### Bark from temperate trees

The tree industry is large in northern European countries, within the temperate climate zone. In Norway, Sweden and Finland, large amounts of coniferous trees (mainly Scots pine, *Pinus sylvestris*, and Norway spruce, *Picea abies*) and smaller amounts of white birch (*Betula pubescens*) are harvested annually, mainly for use as building material and pulp for paper production. This results in large amounts of bark as an unexploited by-product, of which a major part (60%) is used for bioenergy. In 2019, Norway alone had an estimated production of 0.22 million tonnes of wet bark from pine and spruce [96]. Denmark also utilises various fast-growing deciduous trees for bioenergy (e.g. willow, *Salix* spp., hazel, *Corylus* spp., and poplar, *Populus* spp.), and leaves and branches of these species have been used for centuries as winter feed for livestock [57].

Seen in a historical perspective, several tree species have been used as medicine in temperate Europe. Willow bark, various concoctions of pine (bark, needles, and buds) and birch leaves were used for treating urinary tract infections, upper respiratory infections, or mild rheumatism [101]. Similarly, helminth infections of humans or livestock, mainly horses, were traditionally treated with bark or other products of various tree species, e.g. *P. abies*, *P. sylvestris* and juniper (*Juniperus communis*), *Salix* spp., and *B. pubescens* [120]. Willow bark is a source of salicin, a potent anti-inflammatory [63].

The bark of several coniferous species and some deciduous trees growing in the temperate climate zone is rich in CT, predominantly procyanidins (PC) of short to medium mDP. For example, extracts of *P. sylvestris* were reported to contain 3–8% CT comprised of 100% PC with an mDP of 6–7, and *P. abies* contained 1–7% CT comprised of 80–100% PC with an mDP of 7–8 [7]. Similarly, extracts of willow bark contained 14.6% CT with 100% PC and mDP of 4.6 [101]. Other studies have indicated a higher proportion of PD (up to 50%) in various parts of deciduous trees, e.g. hazel, birch, walnut, and catkins and twigs of willow [33].

In general, high levels of anti-parasitic effects *in vitro* from acetone-water extracts of plants have been associated with both a high proportion of PD and a high mDP of the CT within the extract [33, 98]. Similarly, other biological activities of CT, such as *in vitro* immune-modulating activity, is stronger in CT with an mDP > 6 compared to CT with lower mDP [121]. However, several extracts from tree species with high PC levels still had marked anti-parasitic effects [33]. This offers large potential also for the woody species. Consistent with this, *in vitro* activity against ruminant nematodes has been demonstrated for extracts of different parts of a range of tree species, including *Betula*, *Castanea*, *Corylus*, *Pinus*, *Quercus*, *Salix* and *Tilia* spp. [33, 76, 88, 98]; reviewed in reference [48]. More recently, it was demonstrated that acetone: water extracts of bark from *P. abies, P. sylvestris*, and *B. pubescens* inhibited egg hatching of *Teladorsagia circumcincta* by 85–100% [7].

Promising *in vivo* results have been obtained in trials with *Salix* spp. in infected sheep either fed chopped willow branches with leaves [80] or browsing on young willow shoots [36, 82], resulting in a high daily intake of CT. Reduced dag scores (i.e. less soiling of hindquarters) have been found consistently, while reductions in FEC have been observed, although moderate and variable. Examinations of worm burdens *post mortem* have shown reduced levels of abomasal nematodes, particularly *H. contortus*, and small intestinal species, e.g. *Cooperia* spp. [80], or of small intestinal nematodes only [36].

Utilisation of the bark of coniferous species in livestock diets is likely to be increasingly relevant, given its high accessibility. Min et al. (2015) [75] found that feeding pine bark powder in the diet (3.2% CT of diet DM) to goats experimentally infected with *H. contortus* reduced worm burdens by 68% and FEC up to 30 %, without negative impacts on body weight or condition. Furthermore, Wright (2015) [123] found that *H. contortus* infected goats had a lower FEC and worm burden when fed a CT-rich diet consisting of a mixture of loblolly pine (*P. taeda*; native to the Southern USA), bark powder and *Lespedeza cuneata* pellets, compared to separate pine bark and *L. cuneata* diets and a control group fed a diet lacking in CT. These results suggest a synergistic effect between components in *P. taeda* bark and *L. cuneata* when fed in a mixed diet. Moreover, CT in the diet enhanced the immune functions of the host.

Bark extracts have also shown activity against parasites other than nematodes. Teichmann et al. (2016) [111] found that bark extracts from *Salix* spp. inhibited *Cryptosporidium parvum* in cell cultures. More recently, it has been shown that water-acetone extracts of pine bark (*P. sylvestris*) may inhibit *C. parvum* growth in cell culture with an IC_50_ of 25.4 μg CT/mL [12], thus supporting an earlier trial finding reductions in the faecal oocyst counts of *C. parvum-*infected mice offered a commercial pine bark (*P. pinaster*) product [52]. Further, a water extract of *P. radiata* inhibited the sporulation of *Eimeria* coccidia by up to 84% [77]. Anti-cestode effects of pine bark have also been demonstrated in rodents, as Dhakal et al. (2015) [35] found inhibition of *Hymenolepis diminuta* cysticercoid excystation in the same dose-dependent manner as the registered pharmaceutical drug praziquantel. They concluded that PC-rich CT inhibited excystation more effectively than PD-rich CT, which is only partially supported by the above for nematodes. Impacts of bark BP on parasites other than nematodes have yet to be explored *in vivo* in ruminants.

In brief, it seems likely that there is scope for development of anti-parasitic products from the readily available bark of coniferous trees. Despite the current paradigm of PD-rich extracts being the most effective against nematodes, extracts rich in PC may still hold promise. In other parasitic infections, PC-rich extracts may even prove to be superior. However, as bark from the wood industry, particularly conifers, will in most cases have low digestibility and not be practical directly as livestock feed, extraction is necessary in order to obtain a usable product. Extraction with organic solvents, as applied in most experimental studies, is impractical and expensive. Water extraction is most likely the only way forward, as already practised in commercial production of CT from *Acacia* spp. in the tropics [84]. This will probably result in a different composition of CT as well as substantially lower yields. Contamination of bark products with soil and sand is also a major challenge [108]. It is important for future research to address these issues in order to make bark products a viable option for parasite prevention in livestock.

### Bark and wood from tropical and subtropical regions

Global harvest and the production of wood products, mainly wood panels, sawn wood, paper, and paperboard, have risen markedly in the last six decades, and projections show that this trend will continue to increase [51]. Tannins are some of the main by-products derived from wood production globally, which can lead to significant added value through use in the production of leather, oil, wine, beer, ceramic production, and plastics and adhesives; as well as wastewater treatment and other applications [31, 93]. Some 90% of global tannin output consists of CT, and annual production reaches up to 200 000 tons. The tree species used for tannin production vary regionally, being mainly black wattle (*Acacia mearnsii*) in Brazil, South Africa, India, and other tropical countries, and Quebracho (*Schinopsis* spp.) in South America.

Species of *Acacia* are responsible for 9.7% of tropical and subtropical tree plantations. Black wattle (*A. mearnsii*) is the main species grown commercially on an international scale [19, 117]. Wattle tannin is commercially produced by extraction from *A. mearnsii* bark using hot water, with CT as the major component [84]. Ease of extraction and high CT yield makes *A. mearnsii* bark extract an excellent candidate to control GIN in livestock, including ruminants. Its effectiveness *in vitro* has been shown against *H. contortus*, *Trichostrongylus vitrinus*, and *Teladorsagia circumcincta* using the larval feeding inhibition assay [72]. The *in vitro* effect of extract of *A. mearnsii* bark on egg hatch and larval migration is, however, low, with IC_50_ of 2.8 and 12.4 mg/mL, respectively [124]. Ultrastructural changes in the cuticle of *H. contortus* adults have been shown after exposure to tannins [125].

Intake of *A. mearnsii* bark in feed by sheep and goats reduced the GIN FEC [18, 27, 73]. Also, it reduced egg viability and consequently the quantity of L3 from eggs collected from animals fed with *A. mearnsii* bark extract [74]. These results indicate the potential of feeding such tannins to reduce infective larvae on the pasture and, consequently, animal reinfection. Also, numbers of *T. colubriformis* and *Cooperia* spp. adults were reduced in sheep that received *A. mearnsii* bark extracts [18], but they did not reduce *H. contortus*, *T. colubriformis*, and *Oesophagostomum columbianum* adult worms in goats [27]. It has also been shown that the use of *A. mearnsii* bark did not alter blood parameters or carcass measurements of sheep and goats naturally or experimentally infected with nematodes [18, 27, 73, 74].

Supplementary feeding of CT from *A. mearnsii* has been shown to reduce methane emission in cows and sheep [2, 3, 32]. However, this reduction was not observed in sheep infected with *T. colubriformis* and *H. contortus* [60]. *Acacia mearnsii* supplementation also affected the structure of the ruminal microbial community, modulating important microbe groups affected by GIN infection in lambs [25]. Bioactivity to suppress methane production, if carried out in the field, could increase the beneficial effects of feed intake of *A. mearnsii* on animal production.

Quebracho is one of the most important bark extracts used in the tannin industry, mainly in Latin America, where it comes from barks of different trees of the genus *Schinopsis* [50]. The CT concentration ranges from 35% to 73% depending on quebracho extracts [5, 6, 8, 61]. The CT of quebracho are usually described as profisetinidins. The high production and concentration of CT make quebracho extracts an excellent candidate to use in the field as nutraceuticals for ruminants.

Quebracho extracts have shown activity against *H. contortus*, *T. circumcincta*, and *Tr. vitrinus* using larval migration and larval development assays [5, 6, 43]. Results *in vivo* showed efficacy in sheep infected by *H. contortus*, *T. colubriformis*, and *Nematodirus battus*. However, trials are contradictory in the apparent mechanism of action, with reduction of egg output being the more substantial result [5, 6, 71]. In goats, the effect of administration of quebracho extracts on *T. colubriformis* and *T. circumcincta* egg excretion was shown, but not on *H. contortus* [87, 89, 90]. Quebracho supplementation also reduced the excretion of *Eimeria* oocysts in lambs and goat kids, but did not reduce clinical signs of coccidiosis [1]. These results show the importance of more studies with other parasites as potential targets of CT. Evaluation of possible impacts on specific and non-specific immunological responses is an important area for future research.

Tannins from the bark of tropical trees have therefore been shown to have potential use to control GIN in ruminants, and should be considered for use as nutraceuticals to improve livestock production. It is essential to highlight that several factors could influence outcomes of CT feed supplementation from these and other sources, especially dosage, and nematode and host species. The cost of CT sources in different regions should also be considered before planning the use of these compounds as nutraceuticals in livestock. The use of by-products with a high concentration of CT has the potential to make livestock production more sustainable, given the low residues in meat and milk and low environmental impact.

### Tropical by-products other than bark

Several polyphenol-rich materials from temperate regions have been proposed, aiming to achieve added value materials currently considered waste that needs to be discarded in a way that does not affect the environment. A similar approach has been proposed for tropical regions. However, this search has also been prompted by a recent discovery: an *H. contortus* isolate from Mexico showing lower susceptibility to polyphenol-rich acetone: water extracts produced from leaves of plants commonly ingested by local sheep or goats [16, 21, 115]. These authors proposed that the *H. contortus* isolates obtained from animals browsing the tropical forest of Mexico showed some level of adaptation to plant polyphenols, possibly due to the constant exposure of the local GIN to the polyphenol-rich vegetation consumed by the hosts browsing this area.

This finding stimulated the quest for polyphenol-rich plant materials that can be used as ruminant feeds, but which are not included in the normal diet of grazing ruminants. The search began by testing polyphenol-rich plant materials that are not part of the normal diet of ruminants, such as mangrove leaves and locally available AIBPs such as *Coffea arabica* spent coffee grounds (SCG) or *Theobroma cacao* pod husks and leaves. These materials were confronted with a second criterion: the chemical composition of the tested AIBPs should be suitable for ruminant nutrition. A third criterion consisted of the acceptable consumption of the respective AIBPs by ruminants. These three criteria were met both for the SCG and the *T. cacao* leaves [58, 85, 86]; García-Ceballos et al., 2018 [37, 64].

### Evidence of anthelmintic activity of spent coffee grounds (SCG)

The *in vitro* evidence of AH activity against *H. contortus* eggs and L3 larvae of different SCG varieties is shown in Table 1. Different tests showed activity using the egg hatch test, with promising results [58], although poor results were found for eggs in other studies [115]. The larval exsheathment inhibition test showed variation in the activity against L3 when using acetone: water extracts obtained from different SCG varieties from Mexico. The *in vitro* activity also varied when using different *H. contortus* isolates, with stronger activity observed for parasites of French origin, and lower activity for parasites from Yucatán, Mexico [116].

**Table 1:** *In vitro* anthelmintic activity of different varieties of spent coffee ground (SCG) acetone: water extracts against *H. contortus* eggs and L3 of different origins. The condensed tannin content of each extract (catechin equivalent) is also included, as well as the confirmation of the role of polyphenols in the *in vitro* activity by using the tannin inhibitor polyvinylpolypyrrolidone.

Varieties of Mexican *Coffea arabica*	*In vitro* test	*Haemonchus contortus* isolate	Condensed tannin content (%)*	AH activity (% inhibition)	Effective concentration 50% (95% confidence interval) in μg/mL	Confirmed role of polyphenols	Reference
Tazza^®^ Veracruz	EHT at 2400 μg/mL	CENID-INIFAP	5.58	91.6		Not tested	[58]
Tazza^®^ Oaxaca			6.33	53.7			
Tazza^®^ Chiapas			5.89	91.6			
Garat^®^			2.02	92.8			
Garat^®^ Décaf			6.20	94.9			
Inter^®^ Décaf			1.00	87.3			
Starbucks^®^ SG			2.66	92.4			
Starbucks^®^ CS			2.28	57.7			
Garat^®^	EHT	CENID-INIFAP	2.02	No activity		PVPP	[115]
Garat^®^	LEIT at 1200 μg/mL	FESC-UNAM	2.02	93.9		PVPP	[116]
		CENID-INIFAP		98.0			
		Poxila		86.0			
		INRA		100.0			
		Sheep		100.0			
Starbucks^®^ CS	LEIT	FESC-UNAM	2.28		266.56 (221.86 – 309.91)	PVPP	[29]
Starbucks^®^ SG			2.66		176.1 (108.55 – 239.99)		
Starbucks^®^ CS	LEIT	Paraiso	2.28		699.28 (571.31 – 871.04)	PVPP	[85]
			2.66		746.11 (471.35 – 879.44)		
Starbucks^®^ SG							

* Catechin equivalent.

The *in vivo* evidence of AH activity against *H. contortus* or natural GIN infections in sheep and goats consuming different quantities of SCG is shown in Table 2. From the generated evidence, it was possible to confirm that sheep and goats are able to consume a considerable quantity of SCG mixed in the concentrate feed used by these animals (from 10% to 40% of the concentrate feed). Goats were able to consume more SCG as it was possible to reach up to 40% of the concentrate feed. Meanwhile, the studies with sheep could only include 10% of diet. The latter is consistent with recent studies showing a greater ability of adult goats to eat more polyphenol-rich materials compared to adult sheep [41, 118] and the same was recently found for weaned animals [49]. The study with naturally infected goats showed non-significant FEC reductions (25–27% compared to the control diet) after three weeks of ingestion of feed containing SCG [58]. The second study by Palomo-Couoh et al. (2012) [86] showed that *H. contortus-*infected goat kids consuming SCG for 4 weeks had reduced FEC by 69%, and female worm fecundity by 51% compared to kids consuming the control diet. One study with sheep reported a significant reduction of FEC and worm fecundity [28] but in a second study, no AH activity was found although the same quantity of SCG was applied as in the first sheep trial [85]. The difference in the results between the studies could be associated with the *H. contortus* isolate used. The first study used the FESC-UNAM isolate, which is susceptible to polyphenols, and the second study used the polyphenol-resistant Paraiso isolate [20].

**Table 2:** *In vivo* anthelmintic activity against gastrointestinal nematode infections using different types of spent coffee ground (SCG) mixed within the feed of goats and sheep. The condensed tannin content of each extract (catechin equivalent) is also included, as well as the confirmation of the role of polyphenols in the *in vivo* activity using polyethylene glycol.

Varieties of Mexican *Coffea arabica*	Animal species	Gastrointestinal nematode infection	Condensed tannin content of SCG	Inclusion level of SCG	Activity	Confirmed role of polyphenols	Reference
Garat^®^	Goats	Natural mixed infection	2.69%	15%	25% EPG reduction	Not tested	[58]
				30% of Concentrate	27% EPG reduction (non-significant)		
Garat^®^	Goats	FMVZ-UADY	5.84%	40% of concentrate	69% EPG reduction*	PEG	[86]
					51% fecundity reduction		
Starbucks^®^ CS	Sheep	FESC-UNAM	19.6%	10% of diet	25% EPG reduction*	PEG	[28]
Starbucks^®^ SG					26% fecundity reduction*		
Starbucks^®^ CS	Sheep	Paraiso	19.6%	10% of diet	No EPG reduction	PEG	[85]
Starbucks^®^ SG							

* Significant at *p* < 0.05.

### *Evidence of AH activity of* Theobroma cacao *leaves and pod husks*

An early study reported that extracts obtained from *Th. cacao* pod husks and seed husks (seed peels) displayed limited ovicidal activity (eggs remaining as morula) against *H. contortus* eggs from a dose of 600 μg/mL or 2400 μg/mL, respectively. However, that same study also showed that the main activity against eggs consisted of blocking the hatching of larvae formed within eggs. Such activity was described as larvae failing hatching [115]. A second study evaluated the *in vitro* AH activity against *H. contortus* eggs using respective acetone: water extracts obtained from the leaves and pod husks of three Mexican *Th. cacao* varieties. The AH activity against eggs was more evident for the FESC (polyphenol-susceptible) *H. contortus* isolate, with EC_50_ values < 442 μg/mL for husk and leaf extracts. Meanwhile, the activity against eggs for the Paraiso (putative polyphenol-resistant) isolate ranged from 2172 to 700.2 μg/mL in the husk extracts, and from 1115.5 to 684.8 μg/mL in the leaf extracts [65]. The use of PVPP showed that the activity of the *Th. cacao* extracts against eggs was not associated with the polyphenols contained in extracts. Thus, the PSM compounds responsible for the activity against eggs are yet to be elucidated.

The same pod husk and leaf extracts from different *Th. cacao* varieties were also used to evaluate their exsheathment inhibition activity against *H. contortus* L3 [65]. The EC_50_ values of husk extracts ranged from 630.5 to 554.0 μg/mL in the FESC isolate, and from 1068.3 to 472.7 μg/mL in the Paraiso isolate. Meanwhile, the EC_50_ values of leaf extracts ranged from 333.7 to 264.2 μg/mL in the FESC isolate, and from 250.2 to 192.9 μg/mL in the Paraiso isolate. These results clearly show that the *Th. cacao* extracts have stronger exsheathment inhibition activity compared to the activity against eggs of *H. contortus*. Besides, the use of PVPP suggested that the exsheathment inhibition was not associated with polyphenols for pod husk extracts of Azteca and Calabacillo varieties.

The first *in vitro* evidence of AH activity against *Trichostrongylus colubriformis* was recently obtained using acetone: water *Th. cacao* leaf extracts [64]. The Calabacillo and Azteca extracts showed similar activities (EC_50_ = 335.8 μg/mL, CI 95% [309–362.6] and EC_50_ = 387.3 μg/mL, CI 95% [352.5–422.2], respectively). Meanwhile, the Ceylon extract showed the best exsheathment inhibition activity (EC_50_ = 185.6 μg/mL, CI 95% [165.8–205.4]). This study confirmed that the *Th. cacao* leaf extracts showed good AH activity against *T. colubriformis*. Variation in activity suggests that some *Th. cacao* varieties could provide better activity than others, and that should be considered when testing under *in vivo* conditions.

A recent study determined the *in vivo* nutraceutical value of *Th. cacao* leaves against natural GIN infections of goats [37]. The *Th. cacao* leaves (Criollo variety) were harvested and dried indoors at room temperature. The experimental goats consumed the dry *Th. cacao* leaves (TCG) for 9 days and a control group (CG) consumed a conventional diet. The animals consuming the *Th. cacao* leaves were purposely hosting larger natural GIN infection (mean of 2228 ± 28 EPG), while the CG hosted milder GIN infection (mean of 709 ± 9 EPG). The inclusion of *Th. cacao* leaves did not affect the productivity of goats, which were able to consume > 300 g DM of leaves/day. Such high consumption displayed by TCG goats did not affect packed cell volume or live weight, but reduced FEC by 78% compared to CG goats (*p* < 0.05). Thus, *Th. cacao* leaves were confirmed as a nutraceutical candidate against natural GIN infections in goats.

### The different modes of exploitation of AIBPs of tropical origin

The idea behind the use of AIBPs obtained from *Th. cacao* and *C. arabica* may follow very different paths. We propose using the leaves and pod husks that are produced by *Th. cacao* farmers, which could represent added value for these two AIBPs of *Th. cacao*. Thus, the proposal built around the AIBP of *Th. cacao* should directly link cocoa farmers with ruminant farmers. On the other hand, our proposal around *C. arabica* refers to the use of the SCG, which is the by-product that results from the brewing of ground coffee at the smallest scale (home brewing), at coffee shops worldwide, or even the possibility of using this material at an industrial scale where instant coffee is produced. The *C. arabica* SCG approach could also be applicable to small-scale farmers who may use their own coffee waste brewed at home, or may involve linking the AIBP of coffee shops or industries with a large sheep or goat farm, or with groups of small to medium size farmers that could share the product in a more standardised manner.

## Conclusions

The selection of the different AIBPs for this review aimed first at illustrating the worldwide availability of tannin-containing by-products corresponding to the worldwide distribution of GIN in ruminants. Besides this first objective, the different selected examples also aimed to illustrate the diversity of mode of applications of these resources as nutraceuticals, depending on whether they have some nutritional value, and on voluntary feed intake by sheep, goats or cattle. There are various options for delivery of AIBP to livestock, namely:

- direct consumption of the AIBP as potential nutraceuticals;
- combination of the AIBP with a matrix of fodders (tannin-containing or tannin-free pellets);
- extraction of pure active compounds from the AIBP.

To these different options correspond different pros and cons for each AIBP. These are briefly summarised in the following general SWOT analysis, which is proposed based on the currently available information for the selected AIBP. A wide variety of AIBPs have been examined for antiparasitic activity or are subject to investigations in progress; for example, different by-products of the wine industry of olive and/or citrus production, although polyphenols are probably not the sole PSMs involved in the bioactivity. A more general SWOT analysis could therefore summarise the general pros and cons of AIBPs depending on the various modes of use and according to both basic and applied research.

- *Strengths*

As illustrated in this review, the proof of concept of AIBPs as nutraceuticals with anthelmintic activity has been applied worldwide. This is associated with the worldwide concomitant distribution of both GIN and tannin-containing resources.

Overall, AIBPs represent potential low-cost, locally available resources containing bioactive tannins and other polyphenols, which can be proposed to ruminants and can be used directly by farmers. One of the main advantages of AIBP for any potential AH activity is to offer the opportunity to characterise them by measuring CT and to evaluate their bioactivity (e.g. potential AH activity in simple *in vitro* assays) before use as feed supplements. In the case of direct use as nutraceuticals, an evaluation of the nutritional values is also feasible (e.g. for carob, cocoa). Another obvious main advantage for the agro industries generating the by-products is to add value to resources that are usually considered waste and otherwise represent a cost for these industries.

In some circumstances (e.g. coffee, cocoa or carob), AIBPs offer options for organisation of short-circuits of use and/or commercialisation, possibly involving local enterprises. For example, in the case of tropical resources, the manures of sheep and goats can represent a source of fertilizers for coffee and cocoa cultivation, closing the loop. In this respect, the potential use of AIBPs in ruminant (or other livestock) production is an example of the circular economy in a context promoting an agro-ecological approach of animal production, with lower reliance on chemical inputs and better utilisation of locally available resources. Overall, AIBPs represent a model to the circular bioeconomy, one of the main ways to transform our economies and avoid exceeding the earth’s biophysical limits [81].

With regard to further basic research, the diversity of AIBPs represent a wide range of tannins and related compounds, with a diversity of structures. This should allow us to better understand the modes of action of these polyphenols against worms, including potential synergistic interactions (Klongsiriwet et al. 2015 [52]) as well as their possible effects on host digestive physiology, including the gut microbiota and local immunity [121, 122]. The synergistic interactions already shown by enhanced activity of CT derived from multiple plant species, also suggests that the evolution of resistance to active compounds by parasites is either unlikely, or in the case of the apparent adaptive resistance discussed above, overcome by a change of PSM source.

- *Weaknesses*

In the hypothesis of being used as nutraceuticals either directly or by incorporation in some other vegetal matrix (e.g. pellets of fodders to ensure an appropriate overall nutritional level), one of the main issues to solve for the future use of AIBPs relates to their acceptability by the different ruminant species. Palatability of the resources and, ultimately, lack of toxicity, are the first criteria to fulfil before being able to consider any health-promoting effects. In the case of incorporation of AIBPs in a matrix, the addition of flavours to stimulate voluntary food intake is an option. In the case of local use by farmers of non-traditional resources, one weakness could be related to reluctance to adapt to this novel proposed control tool. This could require some early involvement of advisors to explain the aim and to illustrate the potential benefits of incorporating AIBP in animal feed.

In the case of involvement of industrial companies (whether small or large), one weakness is the need to evaluate the economic sustainability of the market and the possible hurdles to organise the collection, preparation, storage, and distribution of the AIBPs at different scales, which might require some investments. The lack of understanding of economic viability of AIBP commercialisation by the feed-additive industry will require assessment of new potential value chains based on local circular approaches and comparing these with current approaches. It will also need to assess and compare costs, benefits and feasibility of using raw AIBP versus extraction of the active components that would increase quality and commerciality, but increase production costs.

The technological processes applied to prepare the different modes of use of AIBPs will have to preserve the bioactive compounds. In addition, ensuring constant quality and stability of the resources will also be one main issue to protect the health of animals but also consumers. For example, as illustrated with chestnut by-products, it has been shown that the extraction of the AIBP by technological processes, whether involving water or not, has major influences on BP quality and on future storage and conservation, e.g. propensity to contamination with fungi, moulds and potential mycotoxins. In the case of coffee and cocoa BP, as well as bark, potential contamination with soil and sand should also be considered. Last, in the hypothesis of AIBPs being considered as resources to extract purified tannins, the nature of the applied solvents can also represent a risk to evaluate, e.g. the use of organic solvents is very difficult if not impossible to handle in large-scale extraction.

It is also increasingly clear that different plant varieties, as well as other factors such as growth conditions, can affect PSM concentration and form, and consequently bioactivity. Variation due to the extraction method adds to this, such that it is difficult to ensure standard quantity and quality of PSM content. Quality control of CT and other PSM extracts from plants destined for use as nutraceuticals is a challenge and potential weakness, in general and also for AIBPs.

Finally, AIBP must be delivered to the animal, and this can be difficult during periods of grazing, which are also generally the times of maximum GIN challenge. Finding ways to align AIBP supplementation with parasite epidemiology to maximise impacts on parasite populations and their impacts on animal performance could be challenging in some livestock systems, especially those involving extensive grazing.

- *Opportunities*

The possible use of natural bioactive compounds to control the main parasitic infections in grazing ruminants correspond to a general public demand to reduce the use of synthetic chemicals in agriculture, and also to general promotion of organic farming systems, especially in the European Union and partner countries.

Besides the possible effects of CT-containing forages in disrupting GIN biology, the various CT-containing legumes have also been associated with other beneficial impacts within the agro-ecological context of livestock production (Mueller-Harvey et al., 2019 [78]). Namely:

- Some other health benefits, in particular in relation to the health of the digestive tract: e.g., prevention of bloat in ruminants and also anti-coccidial effects either in ruminants [15, 105] or other species. For example, some *in vitro* activity against *Cryptosporidium parvum* have been shown with extracts of pine bark [12]. Also, in rabbits, the incorporation of chestnut BP combined with tannins of quebracho, have been associated with some anti-coccidial effects [91] confirming previous results obtained with sainfoin [59]. In pigs, the incorporation of chestnut BP has also been associated with prevention of diarrhoea [39]. Last, anti-inflammatory effects in the gut have been associated with some PSMs [4, 122].
- To limit the environmental consequences of ruminant production by limiting greenhouse gas emissions, including CH_4_ and NO_2_. For example, the incorporation of hazelnut BP in the basal feed of lambs was compared to sainfoin. With both resources, the results showed lower rumen fermentability, and CT decreased CH_4_ production and protein degradability [83]. Increased supply of digestible protein has additional positive environmental outcomes by limiting reliance on feed supplementation, for example from importing soy and other protein-rich feeds that increase demand for land in sensitive areas and feed-food-wood competition; and by limiting reliance on fertilisers.
- Quality of animal-derived products, including meat and milk [38, 95]. The effects of feeding hazelnut peels on dairy ewe performances and milk quality were recently examined [17], suggesting some favourable effects on milk composition for human health.

- *Threats*

**Figure 2:**
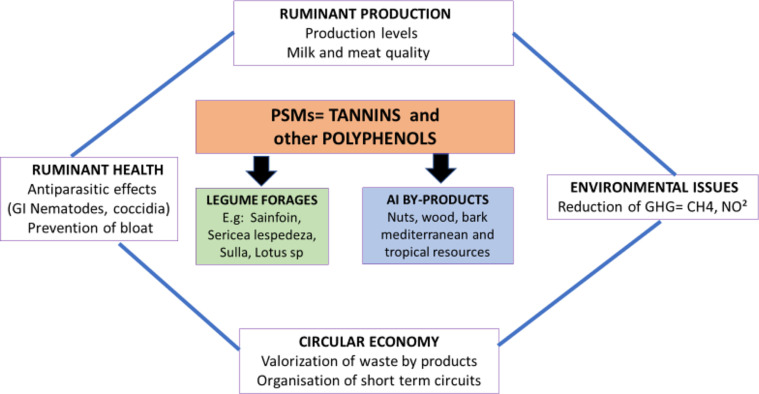
A summarised diagram of the various potential benefits of tannin-containing forages (e.g. sainfoin or sericea lespedeza) and/or by-products in ruminant production in the context of agro-ecological production. Examples and results by referring to the general framework and objectives presented in Figure 1.

Three main threats can be identified which challenge the potential use of tannin-containing AIBP for the nutrition, health and production of livestock, in particular ruminants (Fig. 2).

For the moment, most of the current studies on these bioactive resources rich in natural PSMs have focused on efficacy against gastrointestinal parasites (nematodes and/or coccidia). However, there is a lack of studies on the fate of residues in animal products, either of the PSM that is the source of the benefits or of other co-occurring compounds in the source plants, including toxins and heavy metals. The issue of the regulation of these resources (as additives, complementary feeds, or veterinary medicinal drugs) can strongly affect the future development of such AIBPs. These issues also suppose the development and validation of methods to measure maximum residual values for plant bioactive compounds.

In the circular bio-economy, competition may arise between the use of by-products for several purposes. There is a general trend to identify polyphenols with anti-oxidant properties, and benefits for human health. While multiple benefits of AIBPs are encouraging for their utilisation in the circular economy, this could create competition for their use and drive up prices, reducing economic viability as animal feed. However, pharmaceutical aims may be considered the most valuable use of AIBPs [11].

Lastly, as mentioned in the section on coffee and cocoa, the question of the possible development of resistance by GIN to tannins and polyphenols is pending. Considering the long-standing interactions between hosts, parasites and PSMs from an evolutionary perspective, adaptation of GIN to key PSMs is to be expected. On the other hand, a current hypothesis on the mode of action of tannins and polyphenols on the different key stages (Egg, L3 and adult worms) of GIN seems pleiomorphic based on tannin-protein interactions [45]. This can limit the risks of development of resistance to polyphenols in worm populations. However, this hypothesis needs to be challenged and confirmed by further basic studies.

## Conflict of interest

The authors confirm that they do not have any conflict of interest.

## Notes

**Cite this article as**: Hoste H, Meza-OCampos G, Marchand S, Sotiraki S, Sarasti K, Blomstrand BM, Williams AR, Thamsborg SM, Athanasiadou S, Enemark HL, Torres Acosta JF, Mancilla-Montelongo G, Castro CS, Costa-Junior LM, Louvandini H, Sousa DM, Salminen J-P, Karonen M, Engstrom M, Charlier J, Niderkorn V & Morgan ER. 2022. Use of agro-industrial by-products containing tannins for the integrated control of gastrointestinal nematodes in ruminants. Parasite **29**, 10.
